# RNA-seq Profiling Reveals Novel Target Genes of LexA in the Cyanobacterium *Synechocystis* sp. PCC 6803

**DOI:** 10.3389/fmicb.2016.00193

**Published:** 2016-02-19

**Authors:** Ayumi Kizawa, Akihito Kawahara, Yasushi Takimura, Yoshitaka Nishiyama, Yukako Hihara

**Affiliations:** ^1^Department of Biochemistry and Molecular Biology, Graduate School of Science and Engineering, Saitama UniversitySaitama, Japan; ^2^Biological Science Laboratories, KAO CorporationWakayama, Japan; ^3^Core Research of Evolutional Science and Technology, Japan Science and Technology AgencySaitama, Japan

**Keywords:** cyanobacteria, LexA, RNA-seq, *Synechocystis*, transcriptome

## Abstract

LexA is a well-established transcriptional repressor of SOS genes induced by DNA damage in *Escherichia coli* and other bacterial species. However, LexA in the cyanobacterium *Synechocystis* sp. PCC 6803 has been suggested not to be involved in SOS response. In this study, we performed RNA-seq analysis of the wild-type strain and the *lexA*-disrupted mutant to obtain the comprehensive view of LexA-regulated genes in *Synechocystis*. Disruption of *lexA* positively or negatively affected expression of genes related to various cellular functions such as phototactic motility, accumulation of the major compatible solute glucosylglycerol and subunits of bidirectional hydrogenase, photosystem I, and phycobilisome complexes. We also observed increase in the expression level of genes related to iron and manganese uptake in the mutant at the later stage of cultivation. However, none of the genes related to DNA metabolism were affected by disruption of *lexA*. DNA gel mobility shift assay using the recombinant LexA protein suggested that LexA binds to the upstream region of *pilA7, pilA9, ggpS*, and *slr1670* to directly regulate their expression, but changes in the expression level of photosystem I genes by disruption of *lexA* is likely a secondary effect.

## Introduction

The LexA protein in *Escherichia coli* has been well-characterized as the key regulator of the SOS response induced by DNA damage (Butala et al., 2009). Under non-stress conditions, LexA binds to the promoter regions of more than 40 genes involved in the SOS response and represses their expression. When DNA is damaged, LexA undergoes autoproteolytic cleavage upon association with RecA protein activated through binding of single-stranded DNA fragments. As a consequence of auto-cleavage of the Ala84-Gly85 peptide bond carried out by Ser119 and Lys156, LexA loses DNA binding activity, thereby inducing the SOS response.

Genes encoding LexA homologs are highly conserved in bacterial genomes and LexA-dependent transcriptional regulation of genes involved in DNA repair has been reported in various bacterial species (Erill et al., 2007; Butala et al., 2009), indicating that the regulation of SOS regulon by LexA might be a universal adaptation strategy of bacteria to DNA damage. However, LexA homologs in several cyanobacterial species were suggested not to be involved in the typical *E*. *coli*-type SOS regulation. In *Anabaena* sp. PCC 7120, auto-cleavage of the Ala84-Gly85 bond of LexA does not occur at physiological pH even in the presence of activated RecA (Kumar et al., 2015). In the case of *Synechocystis* sp. PCC 6803 (S.6803), LexA lacks the conserved Ala-Gly auto-cleavage site and the serine of the Ser-Lys dyad required for auto-cleavage activity (Patterson-Fortin et al., 2006) and auto-cleavage of LexA in S.6803 has not been reported so far. DNA microarray analysis revealed that LexA depletion did not affect the expression level of genes involved in DNA metabolism (Domain et al., 2004).

The cellular processes regulated by LexA in S.6803 have been implied by studies reporting isolation of LexA as a binding factor to the promoter region of specific genes, such as the *hoxEFUYH* operon encoding bidirectional hydrogenase (Gutekunst et al., 2005; Oliveira and Lindblad, 2005), *crhR* encoding RNA helicase (Patterson-Fortin et al., 2006), and *sbtA* encoding sodium-dependent bicarbonate transporter (Lieman-Hurwitz et al., 2009). Domain et al. (2004) performed DNA microarray analysis of the LexA-depleted strain and found that most of genes affected were previously reported to be regulated by the availability of inorganic carbon (Wang et al., 2004). Kamei et al. (2001) reported that the *lexA*-disrupted mutant of the motile strain of S.6803 (denoted PCC strain) showed non-motile phenotype. DNA microarray analysis revealed that expression of the *pilA* genes encoding the subunits of the type IV pilus-like structure was lowered in the mutant. Although regulation of various cellular processes has been suggested, we currently have still a fragmentary understanding of the function of LexA in S.6803.

DNA microarray analysis has been the most popular methods of genome-wide transcriptome profiling. However, it has been supplanted by RNA-seq analysis in which isolated transcripts are converted into the complementary DNA (cDNA) followed by direct sequence in a massively parallel DNA sequencing-based approach. The advantages of RNA-seq over DNA microarray are its higher resolution and better dynamic range of detecting differential gene expression (Zhao et al., 2014). In order to obtain the comprehensive view of LexA-regulated genes in S.6803, here we performed RNA-seq analysis of the wild-type (WT) strain and the *lexA*-disrupted mutant. The results of RNA-seq analysis indicate that LexA in S.6803 regulates specific cellular functions such as phototactic motility, accumulation of the major compatible solute glucosylglycerol and subunits of bidirectional hydrogenase, and photosynthetic complexes, but not the SOS response. DNA gel mobility shift assay using the recombinant LexA protein suggested that LexA binds to the upstream region of *pilA7, pilA9, ggpS*, and *slr1670* to directly regulate their expression.

## Materials and methods

### Strains and culture conditions

A glucose-tolerant non-motile strain (GT strain) of *Synechocystis* sp. PCC 6803 was grown at 32°C in BG-11 medium containing 20 mM HEPES-NaOH, pH 7.0, under continuous illumination at 20 μmol photons m^−2^ s^−1^ with bubbling of air. The *lexA* (*sll1626*)-disrupted mutant (Δ*lexA*) was grown under the same conditions, except that 20 μg mL^−1^ kanamycin (Km) was added to the medium. Cell density was estimated by measuring OD_730_ using a spectrophotometer (model UV-160A, Shimadzu).

### Generation of the *lexA* (*sll1626*)-disrupted mutant

The coding region of *lexA* (612 bp, from nucleotide 1319330 to 1318719 according to numbering in CyanoBase) was disrupted by insertion of a kanamycin resistance (Km^r^) cassette. The upstream and downstream fragments including the *lexA* coding sequence were amplified by PCR from the genomic DNA of the WT strain using the primer sets lexA-F and Km-lexA-R (for amplification of 404 bp upstream fragment, from nucleotide 1319525 to 1319122) and Km-lexA-F and lexA-R (for amplification of 394 bp downstream fragment, from nucleotide 1318996 to 1318603; Table S1). Km^r^ cassette was PCR amplified from the pRL161 plasmid using the primer set Km-F and Km-R (Table S1). The amplified *lexA* fragments and Km^r^ cassette were fused together by the fusion PCR method (Wang et al., 2002) using the primer set lexA-F and lexA-R. The WT strain was transformed with the fusion PCR product and transformants (Δ*lexA* mutant) were selected in the presence of Km.

### RNA gel blot analysis

Isolation of total RNA by the hot phenol method and RNA gel blot analyses, using DIG RNA Labeling and Detection Kit (Roche), were performed as described previously (Muramatsu and Hihara, 2003). Template DNA fragments for *in vitro* transcription to generate RNA probes were prepared by PCR using the primers shown in Table S1.

### Immunoblot analysis

Total proteins were extracted from *Synechocystis* cells as described previously (Ishii and Hihara, 2008) and separated by 15% (w/v) SDS-PAGE, followed by electroblotting onto PVDF membranes (Immobilon-P; Millipore). Immunodetection was done using a rabbit polyclonal antibody raised against His-LexA recombinant protein. Goat anti-rabbit IgG conjugated to alkaline phosphatase was used as a secondary antibody.

### Determination of pigment contents

*In vivo* absorption spectra of whole cells suspended in BG-11 medium were measured at room temperature using a spectrophotometer (V-650 Spectrometer, JASCO) with ISV-722 integrating sphare. Chlorophyll and phycocyanin contents were calculated from the peak heights of absorption spectra using the equations described in Arnon et al. (1974).

### RNA-seq analysis

RNA-seq analysis was carried out using cultures at OD_730_ = 0.5 and OD_730_ = 1.0 with three biological replicates. WT and Δ*lexA* were inoculated into new media at OD_730_ = 0.1 and incubated for 50 and 80 h, respectively, to be harvested at OD_730_ = 0.5. Similarly, WT and Δ*lexA* were inoculated at OD_730_ = 0.1 and incubated for 70 and 120 h, respectively, to be harvested at OD_730_ = 1.0. Isolation of total RNA by the hot phenol method was performed as described previously (Muramatsu and Hihara, 2003). To eliminate genomic DNA from total RNA samples, each sample was added with DNase I (TaKaRa) and incubated at 37°C for 3 h. Total RNA concentration was measured with Nanodrop 2000 (Thermo Fisher Scientific). The Ribo-Zero Magnetic Kit for Bacteria (Epicentre) was used to remove ribosomal RNA from each sample. Concentration and quality of mRNA samples were examined using an Agilent 2100 Bioanalyzer. TruSeq RNA Sample Prep Kit v2 (Illumina) was used for cDNA library construction, and the libraries were sequenced using the Illumina MiSeq system. 12 samples in total were analyzed using two cartridge of MiSeq Reagent Kit v3 (Illumina).

A total of 64 million reads data was obtained from 12 samples. To quantify expression level of each gene, nucleotide sequences of obtained reads were mapped to the genomic sequence of GT-I strain of S.6803 (Kanesaki et al., 2012) (NC_017038.1; http://www.ncbi.nlm.nih.gov/nuccore/NC_017038) using CLC Genomics Workbench 7.5.1 software (Qiagen). Raw read counts were divided by length of the transcripts and total number of million mapped reads in each sample to obtain reads per kilobase per million (RPKM) values (Mortazavi et al., 2008). TCC package of R software (Sun et al., 2013) was used to detect the differentially expressed genes between WT and Δ*lexA*. A false discovery rate of <0.01 was considered to be significant.

### Overexpression and purification of his-lexA

The coding region of the *lexA* gene was amplified by PCR using the primers lexA-NdeI-F and lexA-XhoI-R (Table S1), containing NdeI and XhoI sites at their 5′ end, respectively. The amplified *lexA* coding fragment was cloned into the pT7Blue T-vector (Novagen), digested with NdeI and XhoI and subcloned into the same restriction sites in pET28a vector (Novagen) to express the LexA protein with an N-terminal 6 × His-tag.

*E. coli* BL21(DE3) harboring the His-LexA expression construct was grown to an OD_600_ = 0.6 in 250 mL of 2 × yeast extract-tryptone (YT) medium containing 20 μg mL^−1^ Km at 37°C and induced with 0.013% of isopropyl β-D-thiogalactoside for 3 h. The cells were pelleted by centrifugation at 5800 g for 2 min, resuspended in 50 mM sodium phosphate buffer, pH 7.4, containing 0.5 M NaCl and 60 mM imidazole, and disrupted by three rounds of sonication with Sonifier 450 (Branson) for 2 min with interval of 1 min on ice. After the removal of whole cells and insoluble material by centrifugation, the soluble protein fraction was filtered through a 0.2 μm filter (DISMIC-25CS; ADVANTEC). His-LexA was purified by nickel-affinity column chromatography using a HisTrap FF crude (GE Healthcare). The soluble protein fraction was applied to the column equilibrated with 20 mM phosphate buffer, pH 7.4, containing 0.5 M NaCl and 60 mM imidazole, washed with 20 mM phosphate buffer, pH 7.4, containing 0.5 M NaCl and 80 mM imidazole, and eluted with 20 mM phosphate buffer, pH 7.4, containing 0.5 M NaCl and 300 mM imidazole. Purified His-LexA was desalted by a HiTrap Desalting column (GE Healthcare). Protein composition was examined by 15% (w/v) SDS-PAGE followed by staining with Coomassie Brilliant Blue R-250.

### DNA gel mobility shift assay

Probes for DNA gel mobility shift assays were obtained by PCR amplification with primers shown in Table S1 using genomic DNA as a template. The 3′ end of the DNA fragment for each probe was labeled with digoxigenin (DIG)-ddUTP by using the terminal transferase method according to the manufacturer's instructions (DIG gel shift kit 2nd generation; Roche). Gel mobility shift assays were performed by using a DIG gel shift kit 2nd generation (Roche) according to the manufacturer's instruction except that 1 mM DTT was added to the reaction mixture.

## Results

### Characterization of the *lexA* (*sll1626*)-disrupted mutant

**Figure 1:**
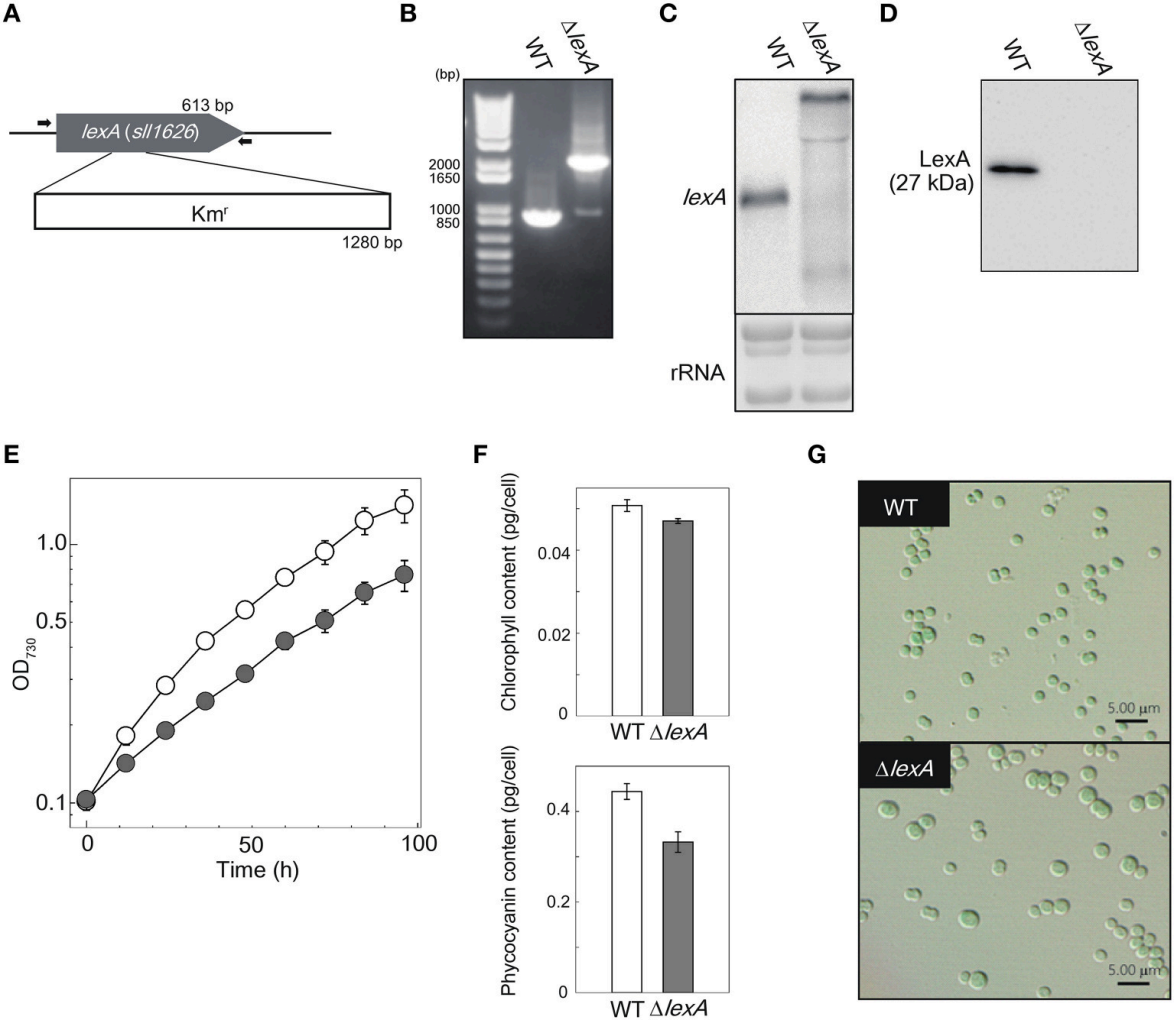
**Generation and characterization of the Δ*lexA* mutant. (A)** Scheme of the construct for disruption of *lexA*. The kanamycin resistance (Km^*r*^) cartridge was inserted into the coding region. Arrows indicate the primers used for PCR amplification shown in **(B)**. **(B)** PCR amplification of the *lexA* gene using genomic DNA from WT and the Δ*lexA* mutant as templates. **(C)** RNA gel blot analysis of the *lexA* transcripts detected by single-stranded RNA probe. 3 μg of total RNA were loaded per lane. Total RNA was stained with methylene blue to show the equal loading. **(D)** Immunoblot analysis of the LexA protein detected by anti-LexA antibody. 5 μg of total protein from cell lysate were loaded per lane. **(E)** Growth curves of WT (open circles) and the Δ*lexA* mutant (closed circles) under normal growth conditions. **(F)** Amounts of photosynthetic pigments calculated from the peak heights of cellular absorption spectra. **(G)** Observation of cell morphology by differential interference contrast microscopy.

To reveal the function of LexA in GT strain of S.6803, we disrupted the *lexA* gene by inserting a Km^*r*^ cassette within the coding region (Figure 1A). Although a fully segregated mutant was not obtained (Figure 1B), RNA gel blot and immunoblot analyses revealed that both the *lexA* transcript (Figure 1C) and LexA protein (Figure 1D) levels were below the detection limit in the partially segregated mutant (Δ*lexA*) grown under normal growth conditions. Under the same conditions, Δ*lexA* displayed several abnormal phenotypes. The doubling time of Δ*lexA* was longer (31.4 h) than that of WT (19.5 h) at log phase, whereas the difference in growth rate between strains became smaller at stationary phase (Figure 1E). Amounts of chlorophyll and phycocyanin in Δ*lexA* calculated from the peak heights of cellular absorption spectra were 93 and 80% of WT levels, respectively (Figure 1F). Microscopic observation revealed that cell size of Δ*lexA* was heterogeneous and tended to be larger than that of WT (Figure 1G).

### RNA-seq transcriptome analysis

**Figure 2:**
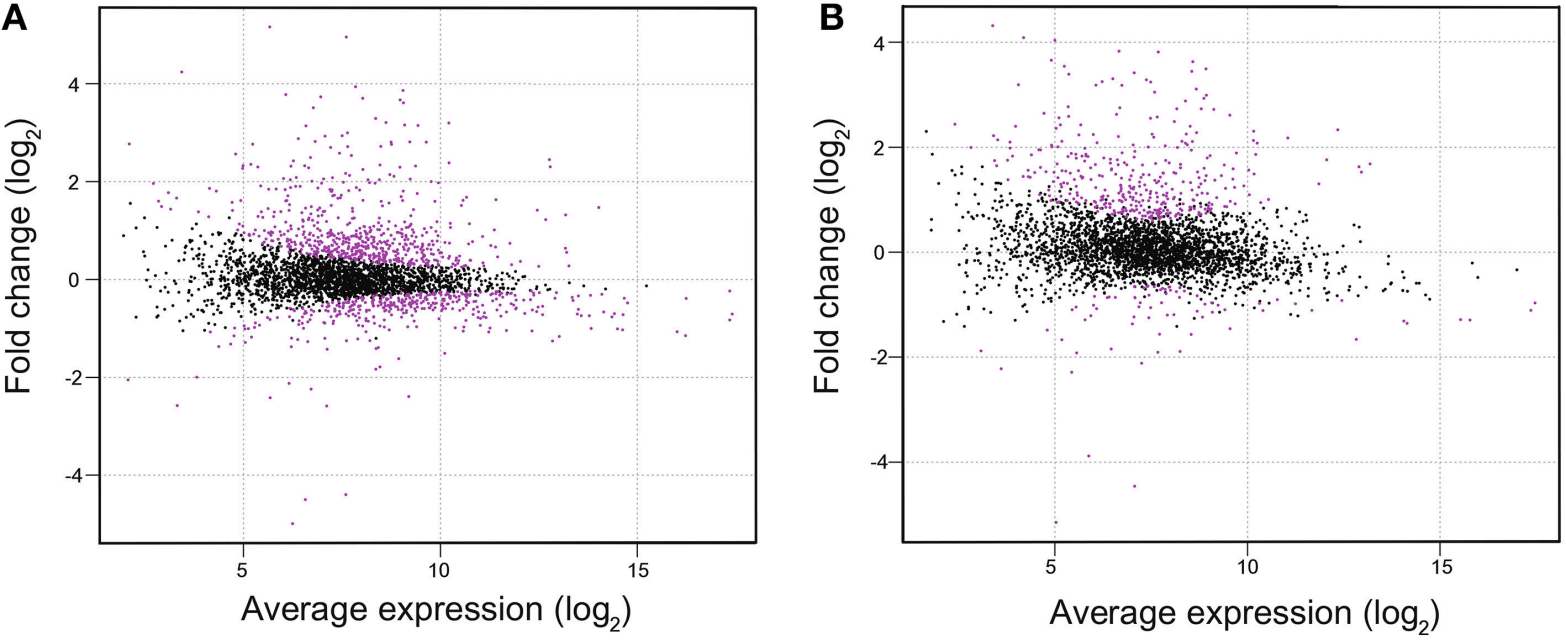
**MA plots of RNA-seq data obtained from WT and Δ*lexA* cells at OD_730_ = 0.5 (A) and OD_730_ = 1.0 (B)**. The MA plot, a scatterplot of log_2_-fold-change (Δ*lexA* /WT) versus average expression in log_2_ scale for each gene, was produced using TCC package. Dots shown in magenta indicate differentially expressed genes with a false discovery rate <0.01.

To investigate the difference in gene expression profile between WT and Δ*lexA*, total RNA was isolated from cultures incubated under normal growth conditions and RNA-seq analysis was performed. Figure 2 shows MA plots of the gene expression data obtained from cultures at OD_730_ = 0.5 and OD_730_ = 1.0. There were 1011 genes differentially expressed between strains at OD_730_ = 0.5 as shown in magenta (Figure 2A). Among them, expression levels of 315 genes were more than two-fold higher and those of 28 genes were more than two-fold lower in Δ*lexA* than in WT (Table S2). In the case of WT and Δ*lexA* cells at OD_730_ = 1.0, there were 447 genes differentially expressed between strains (Figure 2B). Among them, expression levels of 360 genes were more than two-fold higher and those of 21 genes were more than two-fold lower in Δ*lexA* than in WT (Table S3).

Table 1 shows the list of genes whose expression was affected by disruption of *lexA*. The higher resolution and better dynamic range of RNA-seq analysis compared to DNA microarray analysis enabled listing of small ORFs such as *ssl1577, ggpR* (*ssl3076*), *ssr1251, ssr1473* and *ssr3589*, and genes with low expression level (low RPKM value) that cannot be detected by previous DNA microarray analyses. Differentially expressed genes can be categorized into several groups according to related cellular functions as mentioned below.

**Table 1:** **Genes with altered expression in the *lexA*-disrupted mutant**.

**Gene No**.	**Gene symbol**	**Definition**	**OD_730_ = 0.5**			**OD_730_ = 1.0**		
			**Average RPKM**		**Ratio**	**Average RPKM**		**Ratio**
			**WT**	**Δ*lexA***	**Δ*lexA*/WT**	**WT**	**Δ*lexA***	**Δ*lexA*/WT**
**MOTILITY**								
sll1694	*pilA1*	Pilin polypeptide PilA1	7672.37	3764.79	0.49	9711.78	3875.35	0.40
sll1695	*pilA2*	Pilin polypeptide PilA2	245.64	161.17	0.66	272.03	149.86	0.55
slr1930	*pilA7*	Type 4 pilin-like protein	110.72	1163.62	10.51	148.23	1231.28	8.31
slr1931	*pilA8*	Type 4 pilin-like protein	193.59	1186.22	6.13	226.51	1250.18	5.52
slr2015	*pilA9*	Type 4 pilin-like protein	68.30	16.96	0.25	55.96	19.50	0.35
slr2016	*pilA10*	Type 4 pilin-like protein	38.83	23.24	0.60	37.41	16.90	0.45
slr2017	*pilA11*	Type 4 pilin-like protein	72.06	31.47	0.44	73.43	35.82	0.49
slr2018		Unknown protein	84.75	48.63	0.57	97.49	56.12	0.58
sll1291	*taxP2*	Two-component response regulator PatA subfamily	185.08	63.22	0.34	203.50	86.70	0.43
slr1667	*cccS*	Hypothetical protein (target gene of sycrp1)	25.66	13.57	0.53	45.84	18.36	0.40
**GLUCOSYLGLYCEROL METABOLISM**								
slr1670		Unknown protein	29.83	309.81	10.39	24.85	250.51	10.08
slr1672	*glpK*	Glycerol kinase	52.30	299.46	5.73	43.84	227.47	5.19
slr1673	*spoU*	Probable tRNA/rRNA methyltransferase	38.42	179.60	4.67	42.98	144.32	3.36
ssl3076	*ggpR*	Unknown protein	2.03	16.39	8.07	0.00	0.87	*N.D*
sll1566	*ggpS*	Glucosylglycerolphosphate synthase	34.65	497.47	14.36	27.78	431.85	15.54
sll1085	*glpD*	Glycerol-3-phosphate dehydrogenase	32.37	191.71	5.92	36.05	215.11	5.97
slr0529	*ggtB*	Glucosylglycerol transport system substrate-binding protein	17.28	79.09	4.58	18.76	76.80	4.09
slr0530	*ggtC*	Glucosylglycerol transport system permease protein	21.55	102.21	4.74	23.34	88.37	3.79
**HYDROGENASE**								
sll1220	*hoxE*	Diaphorase subunit of the bidirectional hydrogenase	100.28	48.94	0.49	62.85	32.54	0.52
sll1221	*hoxF*	Diaphorase subunit of the bidirectional hydrogenase	64.58	31.77	0.49	49.47	31.90	0.64
sll1223	*hoxU*	Diaphorase subunit of the bidirectional hydrogenase	96.63	49.39	0.51	68.18	47.23	0.69
sll1224	*hoxY*	Hydrogenase subunit of the bidirectional hydrogenase	70.59	35.93	0.51	37.57	28.59	0.76
ssl2420		Unknown protein	54.08	25.39	0.47	42.93	24.51	0.57
slr1675	*hypA1*	Putative hydrogenase expression/formation protein HypA1	31.16	266.71	8.56	33.07	195.50	5.91
**PHOTOSYNTHESIS**								
slr0737	*psaD*	Photosystem I subunit II	9924.04	5201.83	0.52	6914.54	4585.06	0.66
slr1835	*psaB*	P700 apoprotein subunit Ib	34540.96	22842.73	0.66	42976.65	25014.56	0.58
smr0004	*psaI*	Photosystem I subunit VIII	3093.51	2356.63	0.76	290.74	157.92	0.54
ssl0563	*psaC*	Photosystem I subunit VII	10241.74	5794.74	0.57	5317.26	3374.82	0.63
ssr0390	*psaK1*	Photosystem I subunit X	2883.40	1628.37	0.56	1635.89	1152.40	0.70
slr0012	*rbcS*	Rubisco small subunit	3477.08	1936.92	0.56	4542.25	3204.02	0.71
slr0011	*rbcX*	Possible Rubisco chaperonin	3913.65	2224.26	0.57	5157.07	3785.52	0.73
sll0247	*isiA*	Iron-stress chlorophyll-binding protein	55.34	124.36	2.25	53.76	761.15	14.16
sll0248	*isiB*	Flavodoxin	10.09	35.32	3.50	5.91	121.62	20.59
sll1577	*cpcB*	Phycocyanin beta subunit	62726.44	35125.15	0.56	54762.14	27956.67	0.51
sll1578	*cpcA*	Phycocyanin alpha subunit	79538.85	42145.92	0.53	69158.50	35179.81	0.51
sll1579	*cpcC2*	Phycobilisome rod linker polypeptide	13587.17	7921.46	0.58	12570.33	6318.76	0.50
sll1580	*cpcC1*	Phycobilisome rod linker polypeptide	14076.98	8090.37	0.57	12675.97	6193.81	0.49
ssl3093	*cpcD*	Phycobilisome small rod linker polypeptide	4048.58	2772.20	0.68	3023.79	1747.70	0.58
sll1471	*cpcG2*	Phycobilisome rod-core linker polypeptide	847.25	349.04	0.41	638.78	289.60	0.45
ssl2542	*hliA*	High light-inducible polypeptide HliA	22.74	133.52	5.87	26.31	167.07	6.35
ssr2595	*hliB*	High light-inducible polypeptide HliB	71.98	314.95	4.38	40.31	186.15	4.62
slr0506	*por*	Light-dependent NADPH-protochlorophyllide oxidoreductase	247.75	186.82	0.75	318.96	181.53	0.57
slr0749	*chlL*	Light-independent protochlorophyllide reductase iron protein subunit ChlL	489.76	109.67	0.22	98.28	41.69	0.42
slr0750	*chlN*	Light-independent protochlorophyllide reductase subunit ChlN	149.90	49.34	0.33	240.63	81.30	0.34
**CHAPERONES**								
sll0430	*htpG*	HtpG, heat shock protein 90	156.26	589.81	3.77	115.48	607.00	5.26
sll0909	*dnaJ*	DnaJ, heat shock protein 40	24.21	216.45	8.94	22.63	304.56	13.46
sll1514	*hspA*	16.6 kDa small heat shock protein	100.08	1082.85	10.82	121.59	1307.29	10.75
**REGULATORY FUNCTIONS**								
sll0094	*hik37*	Two-component sensor histidine kinase	52.56	33.12	0.63	74.37	39.50	0.53
sll0775		Unknown protein	29.04	391.93	13.50	40.24	339.89	8.45
sll0776	*spkD*	Serine/threonine kinase	16.14	248.58	15.40	27.99	235.49	8.41
sll0777		Putative carboxypeptidase	24.39	219.13	8.98	35.22	210.97	5.99
sll0778		ABC transporter, ATP-binding protein	14.17	72.87	5.14	17.63	71.67	4.07
sll0790	*hik31*	Two-component sensor histidine kinase	49.77	256.96	5.16	74.75	321.59	4.30
sll0797	*nrsR, rppA*	Redox-responsive and/or Ni(II)-responsive regulator, two-component response regulator OmpR subfamily	12.59	7.00	0.56	6.80	10.22	1.50
sll1003	*hik13*	Two-component sensor histidine kinase	10.65	45.77	4.30	10.70	50.35	4.71
sll1626	*lexA*	LexA repressor	1658.80	10652.12	6.42	1451.28	9173.74	6.32
sll1924	*sycrp2*	cAMP receptor protein sycrp1 homolog	23.44	13.60	0.58	11.10	13.47	1.21
slr0895	*prqR*	Transcriptional regulator	15.85	67.22	4.24	19.15	62.39	3.26
slr1564	*sigF*	Group 3 RNA polymerase sigma factor	297.21	217.21	0.73	330.72	209.54	0.63
slr1594	*patA*	Two-component response regulator PatA subfamily	40.38	195.88	4.85	49.36	265.06	5.37
slr1760	*rre8*	Two-component response regulator	15.72	104.05	6.62	26.41	88.06	3.33
slr2098	*hik21*	Two-component hybrid sensor and regulator	26.67	129.37	4.85	38.41	152.72	3.98
**TRANSPORT AND BINDING PROTEINS**								
sll1404	*exbB3*	Biopolymer transport ExbB protein homolog	84.83	131.51	1.55	16.17	290.69	17.98
sll1405	*exbD, sll1405*	Biopolymer transport ExbD protein homolog	28.62	59.22	2.07	13.99	118.68	8.48
sll1406	*fhuA*	Ferrichrome-iron receptor	29.10	57.61	1.98	25.09	132.90	5.30
sll1598	*mntC*	Mn transporter MntC	9.96	28.07	2.82	9.36	112.24	11.99
sll1599	*mntA*	Manganese transport system ATP-binding protein MntA	4.04	17.11	4.24	4.18	65.76	15.74
slr1295	*futA1*	Iron transport system substrate-binding protein	479.40	669.20	1.40	183.65	1131.71	6.16
slr0513	*futA2*	Iron transport system substrate-binding protein	425.93	832.10	1.95	366.04	2076.58	5.67
slr1488		Multidrug resistance family ABC transporter	15.16	46.45	3.06	17.28	130.69	7.56
**OTHER CATEGORIES**								
sll1358	*mncA*	Oxalate decarboxylase, periplasmic protein	248.08	94.87	0.38	133.04	73.57	0.55
sll1688	*thrC*	Threonine synthase	1010.37	704.75	0.70	1408.81	822.45	0.58
sll1709	*gdh*	3-ketoacyl-acyl carrier protein reductase	99.76	504.56	5.06	59.54	418.64	7.03
slr0518	*abfB*	Similar to alpha-L-arabinofuranosidase B	40.86	27.97	0.68	45.82	26.80	0.58
slr0786	*mapB*	Methionine aminopeptidase	6.73	20.91	3.11	6.85	35.69	5.21
slr1204	*degP*	Protease	109.11	511.99	4.69	122.90	572.69	4.66
slr1764	*capA*	Similar to tellurium resistance protein TerE	26.98	222.68	8.25	23.67	125.43	5.30
slr2097	*glbN*	Cyanoglobin	128.40	1919.90	14.95	116.38	1589.74	13.66
slr2116	*spsA*	Probable glycosyltransferase	22.84	11.76	0.51	16.60	14.06	0.85
ssr1720	*tyrS*	Similar to tyrosyl tRNA synthetase	5.26	14.68	2.79	3.77	24.90	6.61
**UNKNOWN PROTEIN**								
sll0249		Hypothetical protein	10.39	23.76	2.29	6.55	84.74	12.95
sll0327		Unknown protein	113.13	1929.23	17.05	157.08	1310.83	8.35
sll0328		Unknown protein	47.79	859.88	17.99	48.45	595.60	12.29
sll0443		Unknown protein	71.82	419.75	5.84	75.08	336.96	4.49
sll0444		Unknown protein	116.90	563.08	4.82	97.11	410.52	4.23
sll0445		Unknown protein	114.46	528.48	4.62	117.45	444.43	3.78
sll0448		Unknown protein	10.17	49.14	4.83	7.21	42.81	5.94
sll0543		Hypothetical protein	677.95	37.76	0.06	535.50	31.01	0.06
sll0783		Unknown protein	84.80	38.09	0.45	86.35	68.05	0.79
sll0846		Hypothetical protein	133.72	577.77	4.32	106.12	532.70	5.02
sll0910		Unknown protein	27.90	188.61	6.76	23.33	243.59	10.44
sll0911		Unknown protein	29.09	170.08	5.85	16.73	124.75	7.46
sll1009		Unknown protein	611.42	3545.01	5.80	926.18	3621.41	3.91
sll1119		Hypothetical protein	109.22	83.03	0.76	73.68	44.88	0.61
sll1236		Unknown protein	30.41	472.85	15.55	27.60	136.10	4.93
sll1239		Unknown protein	88.93	685.38	7.71	41.49	494.27	11.91
sll1240		Unknown protein	23.31	240.78	10.33	18.06	205.72	11.39
sll1241		Unknown protein	26.35	199.16	7.56	14.51	181.65	12.52
sll1247		Hypothetical protein	137.85	61.68	0.45	194.48	104.16	0.54
sll1359		Unknown protein	76.42	37.70	0.49	48.30	36.91	0.76
sll1396		Unknown protein	59.93	13.16	0.22	54.18	13.94	0.26
sll1472		Unknown protein	97.90	48.95	0.50	61.97	49.50	0.80
sll1483		Periplasmic protein	57.32	302.45	5.28	46.76	183.74	3.93
sll1549		Salt-enhanced periplasmic protein	232.67	121.52	0.52	18.94	215.48	11.37
sll1660		Hypothetical protein	45.49	351.04	7.72	48.25	355.40	7.37
sll1722		Hypothetical protein	18.55	130.15	7.01	13.41	45.98	3.43
sll1723		Probable glycosyltransferase	11.63	67.76	5.83	7.87	24.45	3.11
sll1851		Unknown protein	136.11	100.98	0.74	15.16	103.21	6.81
sll1913		Hypothetical protein	24.83	103.47	4.17	18.85	108.19	5.74
sll1921		Hypothetical protein	136.06	1118.71	8.22	152.96	1173.63	7.67
sll1956		Hypothetical protein	68.61	42.42	0.62	60.44	37.54	0.62
slr0105		Hypothetical protein	40.10	327.60	8.17	51.00	312.12	6.12
slr0106		Unknown protein	58.01	324.48	5.59	74.96	308.21	4.11
slr0179		Hypothetical protein	11.14	405.28	36.37	19.47	345.32	17.74
slr0196		Unknown protein	38.61	187.90	4.87	14.43	111.92	7.75
slr0317		Hypothetical protein	18.01	103.04	5.72	20.82	119.39	5.73
slr0362		Hypothetical protein	48.52	240.11	4.95	55.46	204.71	3.69
slr0364		Hypothetical protein	5.58	25.68	4.61	5.73	12.25	2.14
slr0393		Unknown protein	17.69	35.91	2.03	7.26	38.90	5.36
slr0442		Unknown protein	175.06	105.14	0.60	207.58	121.90	0.59
slr0572		Unknown protein	350.05	18.16	0.05	194.88	16.91	0.09
slr0573		Unknown protein	18.80	3.65	0.19	22.65	6.20	0.27
slr0581		Unknown protein	79.35	334.01	4.21	60.26	159.45	2.65
slr0617		Unknown protein	85.17	16.67	0.20	89.51	25.91	0.29
slr0709		Hypothetical protein	88.58	76.13	0.86	96.00	56.95	0.59
slr0868		Unknown protein	20.22	326.40	16.14	13.93	203.19	14.59
slr0869		Hypothetical protein	23.29	185.20	7.95	24.87	165.85	6.67
slr0870		Hypothetical protein	31.36	196.83	6.28	14.82	110.90	7.48
slr0871		Unknown protein	12.74	102.10	8.01	5.49	63.27	11.53
slr1161		Hypothetical protein	306.59	134.80	0.44	251.76	84.91	0.34
slr1162		Unknown protein	131.90	66.56	0.50	104.40	63.19	0.61
slr1278		Hypothetical protein YCF62	32.54	27.87	0.86	78.79	41.89	0.53
slr1484		Unknown protein	48.58	129.12	2.66	29.37	273.35	9.31
slr1485		Salt-induced periplasmic protein	12.48	53.60	4.29	14.24	110.54	7.76
slr1704		Hypothetical protein	162.27	1747.93	10.77	179.40	617.74	3.44
slr1767		Hypothetical protein	39.67	197.11	4.97	19.06	96.15	5.04
slr1772		Probable hydrolase, periplasmic protein	49.33	225.97	4.58	49.08	245.26	5.00
slr1788		Unknown protein	33.57	388.00	11.56	65.92	359.18	5.45
slr1789		Unknown protein	16.30	152.34	9.34	29.57	152.66	5.16
slr1798		Unknown protein	155.94	109.76	0.70	186.54	116.12	0.62
slr1920		Unknown protein	69.46	571.56	8.23	58.59	561.87	9.59
slr2119		Unknown protein	60.74	16.37	0.27	41.99	14.00	0.33
ssl1046		Hypothetical protein	573.18	21.06	0.04	291.05	10.30	0.04
ssl1378		Hypothetical protein	69.57	33.34	0.48	194.48	104.16	0.54
ssl1577		Hypothetical protein	20.16	114.07	5.66	7.78	45.35	5.83
ssr0332		Hypothetical protein	218.38	154.04	0.71	120.96	73.51	0.61
ssr1155		Hypothetical protein	670.58	374.80	0.56	164.79	140.34	0.85
ssr1251		Hypothetical protein	52.57	15.34	0.29	6.04	2.22	0.37
ssr1473		Hypothetical protein	13.23	91.14	6.89	10.36	45.68	4.41
ssr2194		Unknown protein	14.79	614.16	41.52	8.50	181.59	21.37
ssr2615		Hypothetical protein	24.65	17.45	0.71	27.18	9.41	0.35
ssr2962		Hypothetical protein	63.09	276.40	4.38	41.60	191.31	4.60
ssr3570		Unknown protein	61.19	27.67	0.45	30.69	17.30	0.56
ssr3589		Hypothetical protein	19.15	115.47	6.03	9.11	61.37	6.73

#### Motility-related genes

The motile strain of S.6803 exhibits phototactic motility dependent on the type IV-like thick pilus structure (Brahamsha and Bhaya, 2014). In S.6803 genome, there are multiple genes homologous to the *pilA* gene encoding the subunit of the type IV pilus-like structure. Among them, *pilA1* was shown to be responsible for the thick pilus structure, motility, and transformation efficiency (Bhaya et al., 1999; Yoshihara et al., 2001), whereas functions of other *pilA*-like genes are unknown. We observed that their expression is positively or negatively affected by disruption of the *lexA* gene. Expression of *pilA7*-*pilA8* was largely enhanced whereas that of *pilA9*-*pilA10*-*pilA11* and *pilA1*-*pilA2* decreased. The observed decrease in expression level of *pilA1* and *pilA9*-*pilA10*-*pilA11* was consistent with the results of DNA microarray analysis of the Δ*lexA* mutant in the motile PCC strain (Kamei et al., 2001).

Furthermore, we observed that several genes other than *pilA* involved in motility were affected by disruption of *lexA*. Expression of *pixG-pixH-pixI-pixJ1-pixJ2-pixL* (*sll0038*-*0043*) encoding regulatory factors involved in positive phototaxis increased in the mutant (Tables S2). It has been reported that motility is controlled by cAMP level in S.6803 and inactivation of *cya1* encoding adenylate cyclase or *sycrp1* encoding cAMP receptor protein results in loss of motility (Terauchi and Ohmori, 1999; Yoshimura et al., 2002a). Although expression of *cya1* and *sycrp1* itself was not so much affected by disruption of *lexA*, decrease in expression levels of five genes, *pilA9*-*pilA10*-*pilA11-slr2018* and *cccS* (*slr1667*), out of six genes reported to be decreased by disruption of *sycrp1*(Yoshimura et al., 2002b) was observed (Table 1). *cccS* is also considered to be related to motility, since its disruption resulted in loss of the thick pili (Yoshimura et al., 2010). We observed expression level of *sycrp2* is lower in Δ*lexA* (Table 1), although involvement of SYCRP2 in regulation of motility has not been reported.

#### Glucosylglycerol-related genes

In S.6803, glucosylglycerol (GG) is a major compatible solute to adapt to high-salt or high-osmotic pressure conditions (Klähn and Hagemann, 2011). A set of genes related to GG biosynthesis (*ggp, glp*) and uptake (*ggt*) are organized into several gene clusters such as *ggtBCD* (*slr0529*-*0531*), *ggpS-glpD* (*sll1566*-*sll1085*), *ggpP-ggtA* (*slr0746*-*0747*), and *slr1670*-*glpK*-*spoU*-*slr1674*-*hypA1*(*slr1670*-*1675*) in S.6803 genome (Mikkat and Hagemann, 2000; Klähn et al., 2010). RNA-seq analysis revealed that expression levels of these gene clusters were significantly higher in Δ*lexA* than WT (Table 1 and Table S2). Klähn et al. (2010) reported that a small ORF, *ggpR* (*ssl3076*), exists overlapping with the transcription initiation site of *ggpS* and its promoter region. Expression of *ggpR* was also induced by disruption of *lexA* (Table 1).

#### Hydogenase-related genes

Expression level of the *hoxE*-*hoxF*-*hoxU*-*hoxY*-*hoxH* operon encoding subunits of bidirectional NiFe-hydrogenase was lower in Δ*lexA*. This observation is consistent with the previous study reporting that LexA acts as a transcriptional activator for the *hox* operon (Gutekunst et al., 2005). On the other hand, the expression level of the *hypA1* gene involved in hydrogenase maturation increased in Δ*lexA*.

#### Photosynthesis-related genes

In S.6803, photosystem (PS) I complex is comprised of 11 subunits and genes encoding these subunits (*psa*) are dispersed throughout the genome (Kaneko et al., 1996). We found that the expression level of every PSI gene was lower in Δ*lexA* than WT. The expression level of genes encoding subunits of phycobilisome (*cpc, apc*) was also lower in the mutant, whereas the expression level of genes encoding PSII subunits (*psb*) was not so much affected by disruption of *lexA*. Expression levels of *chlL*-*chlN* encoding subunits of light-independent protochlorophyllide reductase and that of *por* encoding light-dependent protochlorophyllide reductase were lower in Δ*lexA*. Both light-dependent and -independent enzymes catalyzing the last step of chlorophyll biosynthesis are likely to be under the control of LexA. On the other hand, expression level of *hliA* and *hliB* encoding high-light inducible proteins was higher in Δ*lexA* than WT.

#### SOS-response related genes

Previous studies suggested that LexA in S.6803 is not involved in the SOS response. Neither *lexA* nor *recA* expression was induced upon UV-irradiation (Domain et al., 2004; Patterson-Fortin et al., 2006) and none of DNA metabolism-related genes was listed as genes induced or repressed by LexA depletion (Kamei et al., 2001; Domain et al., 2004). Similarly, induction or repression of DNA metabolism-related genes by disruption of *lexA* was not observed in our RNA-seq analysis.

#### Genes differentially expressed in Δ*lexA* at the later stage of growth

Several genes expressed under iron-limiting conditions such as *exbB*-*exbD*-*fhuA* operon involved in inorganic iron uptake (Jiang et al., 2015), *futA1* and *futA2* encoding subunits of iron transporter (Katoh et al., 2001), and *isiA*-*isiB* operon encoding iron-stress inducible proteins (Vinnemeier et al., 1998) were highly induced in Δ*lexA* at OD_730_ = 1.0 but not in OD_730_ = 0.5. In the case of *mntA* and *mntC* encoding subunits of manganese transporter (Bartsevich and Pakrasi, 1995), their expression level was already higher in Δ*lexA* at OD_730_ = 0.5 and showed further increase at OD_730_ = 1.0.

### DNA gel mobility shift assay

**Figure 3:**
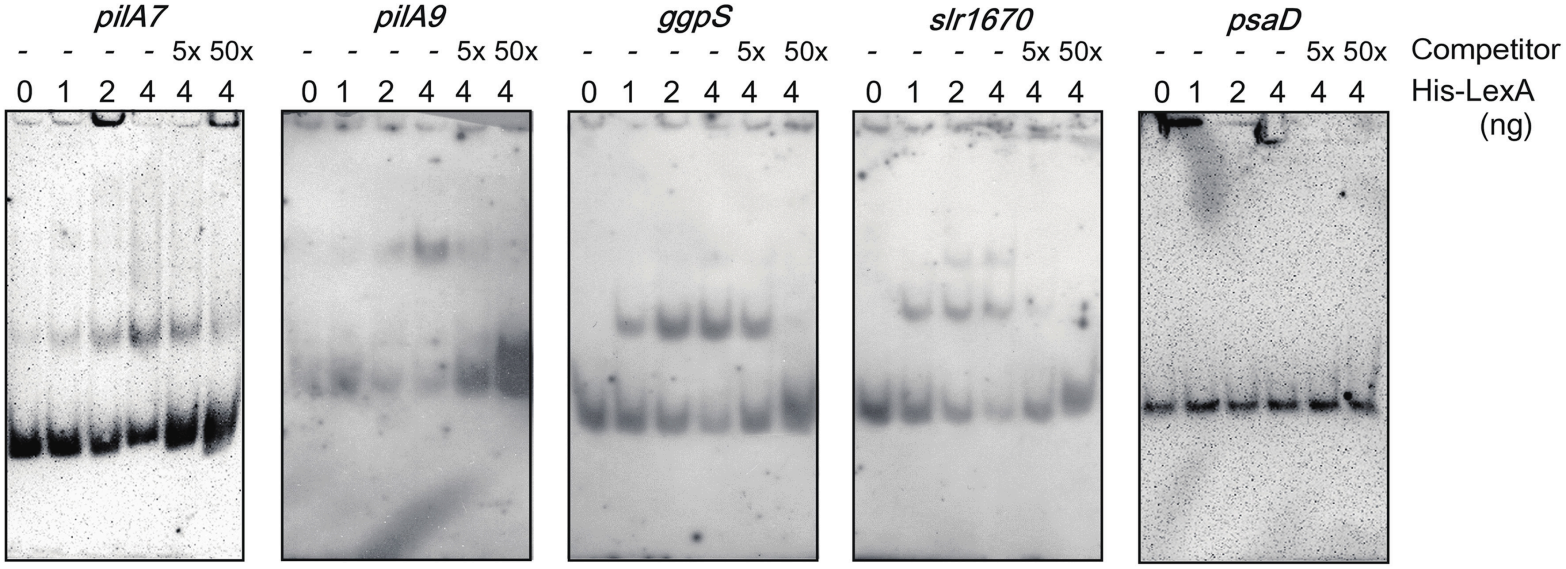
**DNA gel mobility shift assay of the promoter segments of putative target genes with His-LexA**. DIG-labeled promoter segments of *pilA7, pilA9, ggpS, slr1670*, and *psaD* were incubated for 25 min at room temperature with His-LexA added at indicated concentrations. five-fold and 50-fold excess amounts of the non-labeled promoter segments were added as a competitor. Samples were separated on a 6% polyacrylamide gel.

DNA gel mobility shift assay was performed to examine whether LexA directly regulates expression of putative target genes listed by RNA-seq analysis (Figure 3). We observed induction of the *pilA7*-*pilA8* operon and repression of the *pilA9*-*pilA10*-*pilA11* operon in Δ*lexA* (Table 1). Binding of His-LexA to the promoter regions of both operons (for the *pilA7* operon from nucleotide 2222102 to 2222304 and for the *pilA9* operon from nucleotide 755577 to 755778, according to numbering in CyanoBase) was observed, indicating that LexA directly activates or represses expression of these *pilA* operons. We also examined whether His-LexA binds to the upstream region of the two divergently transcribed operons, *ggpS-glpD* and *slr1670*-*glpK*-*spoU*-*slr1674*-*hypA1*, both of which are highly induced in Δ*lexA*. His-LexA bound to the promoter fragment of each operon (for the *ggpS* operon from nucleotide 1949371 to 1949186 and for the *slr1670* operon from nucleotide 1949332 to 1949534). It is notable that LexA-binding site for the the *ggpS* operon is within the coding region of *ggpR* (nucleotide 1949372 to 1949100). Our results suggest that LexA binds to at least two binding site located in the intergenic region of the *ggpS* and *slr1670* operons to repress their expression. Next, we examined the binding of LexA to the upstream region of PSI genes by using light-responsive promoter fragments containing the HLR1 sequence recognized by the response regulator RpaB (Seino et al., 2009). Binding of His-LexA to the promoter region of PSI genes was not observed (Figure 3) or much weaker than that to the *pilA7, pilA9, ggpS*, and *slr1670* promoters and not reproducible. This indicates that decrease in expression levels of PSI genes in Δ*lexA* may be a secondary effect.

## Discussion

### Effects of disruption of the *lexA* gene in S.6803

In this study, we created the gene-disrupted mutant of *lexA* in GT strain of S.6803 to obtain the comprehensive view of LexA regulon by RNA-seq analysis. Although Kamei et al. (2001) successfully obtained the fully-segregated *lexA* mutant from the motile PCC strain, in most cases the Δ*lexA* mutant invariably retained the WT copy of the *lexA* gene (Domain et al., 2004; Gutekunst et al., 2005) and we also could not obtain fully-segregated mutant (Figure 1B). The heterogeneous appearance of the Δ*lexA* mutant cells (Figure 1G) may be caused by difference in the extent of segregation. However, despite the existence of the WT copy of *lexA*, immunoblot analysis revealed that LexA protein level was below the detection limit in our mutant (Figure 1D).

To date, LexA in S.6803 has been reported to be involved in transcriptional regulation of genes related to various cellular functions. Our RNA-seq data are consistent with some of these reports, e.g., positive regulation of the *hox* operon reported by Gutekunst et al. (2005) and positive regulation of the *pilA* genes reported by Kamei et al. (2001). However, we could not observe the large effect of LexA depletion on carbon metabolism-related genes reported by Domain et al. (2004). Domain et al. isolated RNA for DNA microarray analysis from concentrated cultures incubated on plates for 2 h. The growth condition must be largely different from our liquid culture, which may cause the difference in gene expression profile. *in vitro* transcription/translation assay performed by Patterson-Fortin et al. (2006) showed that CrhR protein accumulation decreased in response to increasing LexA concentration. However, in our data, expression level of *crhR* was not affected by disruption of *lexA*.

RNA-seq data in this study suggested involvement of LexA in regulation of (1) phototactic motility, (2) accumulation of GG, (3) bidirectional hydrogenase, and (4) photosystem I and phycobilisome complexes. We also observed increase in expression level of genes related to iron and manganese uptake in Δ*lexA* at OD_730_ = 1.0. LexA may be involved in stage specific repression of these genes, but it is also possible that these genes were upregulated as a consequence of iron and manganese limitation in the mutant culture during prolonged incubation. We will discuss regulation of cellular processes (1)–(4) by LexA in the following sections.

### Cellular processes regulated by LexA in S.6803

#### Phototactic motility

Kamei et al. (2001) reported that disruption of the *lexA* gene in the motile PCC strain resulted in decrease in expression level of *pilA* genes and loss of thick pili and motility. Our RNA-seq analysis showed that expression levels of genes related to phototactic motility are largely affected by disruption of *lexA* also in the non-motile strain. In addition to the decrease in expression level of *pilA1* and *pilA9*-*pilA10*-*pilA11* reported in Kamei et al. (2001), we observed significant induction of *pilA7-pilA8*. Furthermore, expression levels of several genes related to positive phototaxis and cAMP signaling were affected. Although many non-motile mutants were so far isolated from the PCC strain, information on the mechanism of transcriptional regulation of motility-related genes is limited. Bhaya et al. (1999) reported decrease in expression level of *pilA1* and *pilA2* by disruption of the *sigF* gene encoding an alternative sigma factor. Yoshimura et al. (2002b) and Dienst et al. (2008) reported decrease in expression level of *pilA9*-*pilA10*-*pilA11-slr2018* and *cccS-cccP* by disruption of *sycrp1* encoding cAMP receptor protein and *hfq* encoding RNA chaperone homolog, respectively. Panichkin et al. (2006) reported decrease in expression level of *pilA9-pilA10-pilA11-slr2018* and increase in that of *pilA5-pilA6* and *pilA1-pilA2* by disruption of *spkA* encoding Serine/threonine protein kinase. None of these reports showed the direct interaction of these regulatory factors with *pilA* genes and LexA in this study is the first report of binding of transcriptional regulator to their upstream region (Figure 3). Involvement of SYCRP1 in transcriptional regulation of *pilA* genes through the direct regulation of LexA is not likely, since no SYCRP1 binding sequence has been detected in the upstream region of the *lexA* gene (Omagari et al., 2008; Xu and Su, 2009). Further examination of relationship between LexA and previously identified regulatory factors which affect motility may be a key to understanding of signal transduction mechanism regulating phototactic motility.

#### Accumulation of GG

In order to acclimate to high-salt or high-osmotic pressure conditions, S.6803 accumulates the compatible solute GG. Upon a salt shock, genes related to both GG biosynthesis (*ggp, glp*) and uptake (*ggt*) are induced (Kanesaki et al., 2002; Marin et al., 2004). GG is synthesized by a two-step reaction in S.6803. First, condensation of ADP-glucose and glycerol 3-phosphate is catalyzed by GG-phosphate synthase (GgpS) and then the intermediate is dephosphorylated by GG-phosphate phosphatase (GgpP) (Hagemann and Erdmann, 1994). Glycerol-3-phosphate dehydrogenase (GlpD) and glycerol kinase (GlpK) are involved in the metabolism of glycerol-3-phosphate, a precursor of GG. Uptake of GG from the environment is performed by ABC transporter consisting of an ATP-binding protein (GgtA), a substrate-binding protein (GgtB) and two integral membrane proteins (GgtC and GgtD) in S.6803 (Mikkat and Hagemann, 2000). All of these genes are induced by the disruption of *lexA* (Table 1 and Table S2). DNA gel mobility shift assay revealed that His-LexA protein binds to the upstream region of two divergently transcribed operons, *ggpS-glpD* and *slr1670*-*glpK*-*spoU*-*slr1674*-*hypA1* (Figure 3). To date, sigma factors SigF (Marin et al., 2002) and SigB (Nikkinen et al., 2012), a small protein GgpR (Klähn et al., 2010) and a response regulator Slr1588 (Chen et al., 2014) were reported to be involved in transcriptional regulation of the *ggpS-glpD* operon. Our result suggests the existence of the additional regulatory mechanism, namely, repression of the divergent *ggpS* and *slr1670* operons by LexA. Expression of *ggpR* may also be repressed by LexA, judging from the fact its expression was induced by the disruption of *lexA* (Table 1). Salt-stress inducible genes such as *hliA, hliB, hspA, prqR, degP, sll1723, sll0846, sll1483, slr1704, sll1236, slr0581*, and *ssr2194*, reported in the previous DNA microarray studies (Kanesaki et al., 2002; Marin et al., 2004), were also induced by the disruption of *lexA* (Table 1). There is possibility that LexA acts as a repressor for multiple salt-stress inducible genes as well as the *ggpS* and *slr1670* operons.

#### Bidirectional hydogenase

Regulation of the *hox* operon by LexA has been extensively studied in S.6803 (Oliveira and Lindblad, 2009). LexA was shown to bind to two distinct regions of the *hox* promoter, −198 to −338 and −592 to −690, relative to the start codon of *hoxE* (Gutekunst et al., 2005; Oliveira and Lindblad, 2005) and work for positive regulation of hydrogenase activity (Gutekunst et al., 2005). Regulation of hydrogenase-related genes by LexA may be common among cyanobacterial species, judging from the reports on LexA homologs in *Anabaena* sp. PCC 7120 (Sjöholm et al., 2007) and *Lyngbya majuscula* CCAP 1446/4 (Ferreira et al., 2007).

#### Photosystem I and phycobilisome

In the Δ*lexA* mutant, chlorophyll and phycocyanin contents were lower than those in WT (Figure 1F). This may be caused by decreased expression level of genes encoding subunits of PSI (*psa*), subunits of phycobilisome (*cpc, apc*) and both light-dependent and -independent protochlorophyllide reductase (*chlL, chlB, por*). It is known that these photosynthesis-related genes show the quite similar response to the changing light environment (Muramatsu and Hihara, 2012). The response regulator RpaB regulates high-light response of photosynthesis-related genes by binding to their promoter regions under low-light conditions (Wilde and Hihara, 2016). PSI genes and *hli* genes are positively- and negatively-regulated target genes of RpaB, respectively (Kappell and van Waasbergen, 2007; Seki et al., 2007; Seino et al., 2009). Repression of PSI genes and induction of *hli* genes by disruption of LexA (Table 1) seem to suggest overlapping roles of RpaB and LexA in regulation of photosynthetic gene expression. However, clear and reproducible band shift was not observed when binding of His-LexA to the promoter regions of PSI genes was examined (Figure 3). It is possible that changes in expression levels of photosynthesis-related genes in Δ*lexA* are not the consequence of loss of regulation by LexA but a secondary effect.

### Search for LexA binding sites in the target promoters

Our results of DNA gel mobility shift assay suggest that LexA binds to the upstream region of *piA7, pilA9, ggpS* and *slr1670* to directly regulate their expression (Figure 3). To date, several nucleotide sequences for LexA binding site have been identified by DNA gel mobility shift assay, for example, 5′-TTTATTTGA ACTATTTTT-3′, 5′-TTTTTCGTTGTCTAA ATT-3′ (Oliveira and Lindblad, 2005), 5′-CTA-N_9_(AT-rich)-CTA-3′ (Patterson-Fortin and Owttrim, 2008), and 5′-AGT AACTAGTTCG-3′ (Gutekunst et al., 2005) in S.6803 and 5′-TAG TACTAATGTTCTA-3′ in A.7120. (Mazón et al., 2004). However, these LexA binding sequences could not be found in the promoter fragments to which His-LexA bound. Instead, we found that a 5′-TTTTG(A/T)TNAC-3′ sequence commonly exists in these promoter fragments (Figure S1). The sequence is located around the putative transcription start site in the case of the negatively-regulated target genes, *ggpS, piA7*, and *slr1670*, whereas it is located further upstream region in the case of the positively-regulated *pilA9* gene. It has been reported that a certain global transcriptional regulator, such as NtcA and RpaB in S.6803, can act as both repressor and activator dependent on the location of the binding site (García-Domínguez et al., 2000; Seino et al., 2009). Binding of the transcriptional regulator causes repression when its binding site overlaps the RNA polymerase-binding site, whereas activating effect is observed when the binding site is located further upstream. The location of 5′-TTTTG(A/T)TNAC-3′ sequence in four LexA-target promoters seems consistent with the scheme.

### Physiological roles of cyanobacterial LexA

Results of RNA-seq analysis (Table 1) together with DNA gel mobility shift assay (Figure 3) suggest LexA in S.6803 can positively or negatively regulate various cellular processes such as phototactic motility, GG accumulation and hydogenase activity. Regulation of such a wide range of cellular processes by LexA was reported in other bacterial species. For example, the *lexA* mutant of *Clostridium difficile* showed pleiotrophic phenotypes such as filamentous structure due to inhibition of cell division, decreased sporulation, decrease in swimming motility and increased biofilm formation (Walter et al., 2015). In this case, LexA acts as a regulator of DNA damage in addition to the above mentioned biological functions. In contrast, DNA microarray data from different research groups (Kamei et al., 2001; Domain et al., 2004) and our RNA-seq data suggest LexA in S.6803 is not involved in regulation of SOS genes. In S.6803, expression of *lexA* and *recA* was not induced upon UV-irradiation (Domain et al., 2004; Patterson-Fortin et al., 2006). Similarly, in *Anabaena* sp. PCC 7120, expression of *lexA* was not induced upon UV-B exposure or treatment with a DNA damaging agent mitomycin C (Kumar et al., 2015). In these freshwater species, LexA-independent protection mechanism for DNA damage may have evolved and LexA may have become devoted to regulating other cellular processes. Then, what is the physiological meaning of the coordinated regulation of phototactic motility, GG accumulation, and hydogenase activity by LexA in S.6803? We searched for environmental conditions where LexA-target genes are coordinately regulated using CyanoEXpress gene expression database (http://cyanoexpress.sysbiolab.eu/) and found that salt stress causes induction of GG metabolism-related genes and repression of *hox* operon and *pilA* genes in WT (Shoumskaya et al., 2005; Dickson et al., 2012). The expression profile is similar to that observed by disruption of *lexA* (Table 1), indicating the possibility that transcriptional regulation by LexA is temporarily inactivated under salt stress conditions.

Recently, it has been suggested that the SOS response in the marine *Synechococcus* is regulated by LexA like *E. coli* (Blot et al., 2011; Tetu et al., 2013). Cyanobacterial LexA genes can be clustered into three groups, Clade A containing *Gloeobacter violaceus* PCC 7421, Clade C containing marine picocyanobacteria and Clade B containing most remaining species (Li et al., 2010). There may exist high degree of variation of LexA regulons among species belonging to these three clades. By examination of what kind of cellular processes LexA regulates, we will be able to know decision of each species about how to use the transcriptional regulator LexA for better adaptation to changing environment.

## Author contributions

The study was conceived by AYK and YH, with design input from AKK. Experiments were performed by AYK and AKK. Data analysis and interpretation was done by all authors. The manuscript was prepared by AYK and YH, and reviewed by all authors.

## Funding

This work was financially supported by the Core Research of Evolutional Science & Technology (CREST) programs from the Japan Science and Technology Agency (JST).

### Conflict of interest statement

The authors declare that the research was conducted in the absence of any commercial or financial relationships that could be construed as a potential conflict of interest.

## Supplementary material

Table S1 **Oligonucleotides used in this study**.

Table S2 **RNA-seq data of WT and Δ*lexA* at OD_730_ = 0.5**.

Table S3 **RNA-seq data of WT and Δ*lexA* at OD_730_ = 1.0**.

Figure S1 **Consensus sequences for LexA binding site in four LexA-target promoters identified with MEME**.
