# Supplementary material for: RNA-seq Profiling Reveals Novel Target Genes of LexA in the Cyanobacterium Synechocystis sp. PCC 6803
Source: Front Microbiol. 2016 Feb 19;7:193. doi: 10.3389/fmicb.2016.00193 (PMC4759255; doi:10.3389/fmicb.2016.00193)
Supplement: Figure S1 — Consensus sequences for LexA binding site in four LexA-target promoters identified with MEME. [file Image1.PDF]

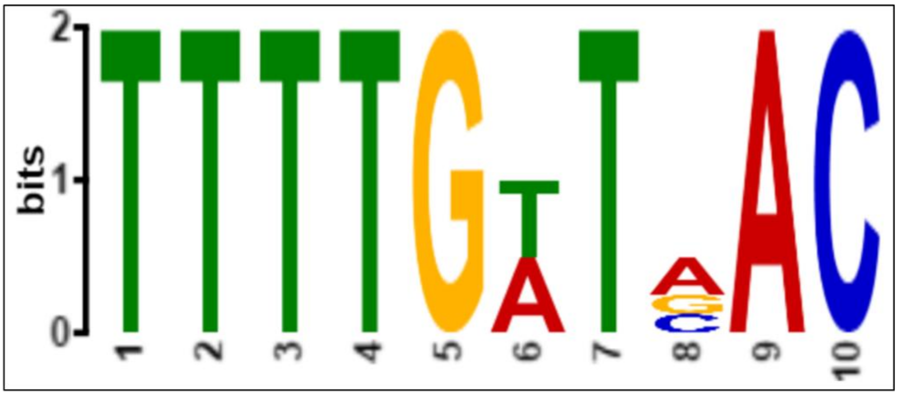

	Position	*p*-value	Sequence
pilA7	2222265.5	1.44e-5	CCACTTGCTT TTTTGATCAC TATAAATATA
pilA9	755580.5	1.44e-5	GTTTAAGTTG TTTTGTTGAC GGCTTTTGTG
ggpS	1949310.5	6.64e-6	ATTCTATTTT TTTTGTTAAC CTTAAAACTA
slr1670	1949435.5	6.64e-6	TTTTATGGAG TTTTGATAAC ATTGAGCTTT

Figure S1. Consensus sequences for LexA binding site in four LexA-target promoters identified with MEME.

Upstream region of LexA-target genes were submitted to MEME (http://meme-suite.org/tools/meme) for prediction of the conserved DNA element. Position means the center of the putative binding sites according to numbering in CyanoBase (genome.microbedb.jp/cyanobase/).
